# Supplementary material for: A multicentre study on spontaneous in-cage activity and micro-environmental conditions of IVC housed C57BL/6J mice during consecutive cycles of bi-weekly cage-change
Source: PLoS One. 2022 May 25;17(5):e0267281. doi: 10.1371/journal.pone.0267281 (PMC9132304; doi:10.1371/journal.pone.0267281)
Supplement: S1 File — (PDF) [file pone.0267281.s001.pdf]

Supporting information

A multicentre study on spontaneous in-cage activity and micro-environmental conditions of IVC housed C57BL/6J mice during consecutive cycles of bi-weekly cage-change

B. Ulfhake, H. Lerat, J. Honetschlager, K. Pernold, M. Rynekrová, K. Escot, C. Recordati, R. Kuiper, G. Rosati, M. Rigamonti, S. Zordan, J.-B. Prins.

Randomisation

At the LUMC, randomisation was achieved using a randomisation table where the allocation to cage numbers was randomly distributed over the consecutive order in which animals were taken out of the transport boxes in which they arrived at the facility. At KI and UGA, delivered animals were dispensed to the study cages by systematic sampling with a random starting point. At IMG, the animals were randomised by the equal selection of active animals and less active animals or animals hiding in corners of the transport box to the different study cages.

Detailed protocol of measurement of NH_3

a) Switch the Dräger ammonia detector on and perform an initial instrument zero-calibration according to manifacturer's instructions.

b) Remove the sealing screw from the front hole of the top cage (order of measurements per cage: front, middle, rear).

c) Insert the entire length of the copper probe through the hole. The cage remains inserted in the DVC® rack and connected to the nozzles (cage remains ventilated).

d) Keep the probe inserted for a minimum of 30 seconds till a stable reading is obtained.

e) Record the value and remove the probe from the hole.

f) Reinstall the seal of the front hole and remove the screw from the next hole.

g) Before inserting the probe ensure that the detector has returned to base-line = room level = 0. If the detector has not returned to zero perform the zero-calibration procedure according to manifacturer's instructions.

h) Take measurements from middle and rear cage areas before moving to the next cage.

Fig. 1

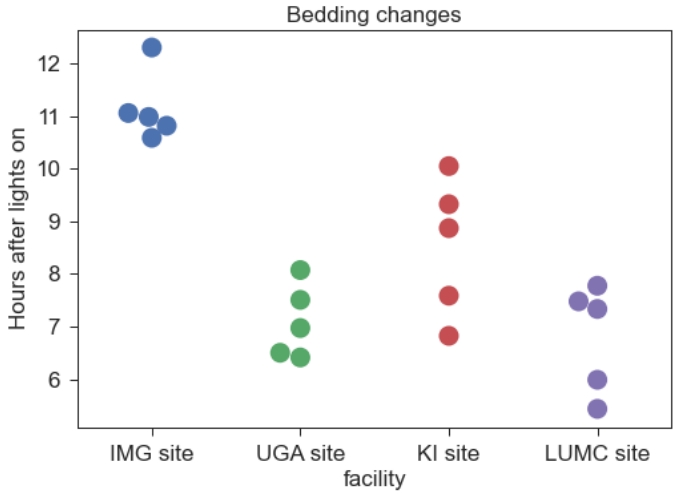

Fig. S1 Time of bedding change. The figure shows the time when each of the 5 cage changes was performed, expressed as hours after lights on at the four centres.

Germ free mice

The mice delivered to KI derived from the 5th generation of a germ-free colony established by hysterectomy (caesarean section) of the strain https://www.jax.org/strain/000664 delivered directly from the JAX lab in 2018.

To establish a new colony according to the routines at the Ulm axenic facility, the build-up of isolator incl. sterilisation, wiping out inside isolator, material import and hygienic control analysis takes approx. 4-6 weeks.

The germfree isolators are maintained in overpressure. In- and outgoing air via HEPA filter. Each isolator holds a maximum of 7 to 9 cages of type II and type II long. Housing density maximum is 4 or 5 mice per cage, respectively, according to ETS 123, appendix A. Cages and drinking bottles are kept inside isolator until isolator is dismounted.

All material going inside an isolator is autoclaved in specific closed cylinders at 134°C. Water is autoclaved in glass bottles at 132°C. Materials that are not autoclavable at these temperatures will be sterilized with peracetic acid (2%). Weekly change of bedding and filling-up water bottles. Food is filled up weekly. Breeding set-up and weaning of mice according to the need.

Bedding: SAFE ® BK 8-15 (spruce wood)

Food: Sniff, V1184-300 autoclavable, mouse breeding, high energy

Temperature inside isolator is 22°C +/- 2°C and relative humidity 55% +/-10%.

With outgoing materials, samples are analysed for germfree condition.

Fig. 2

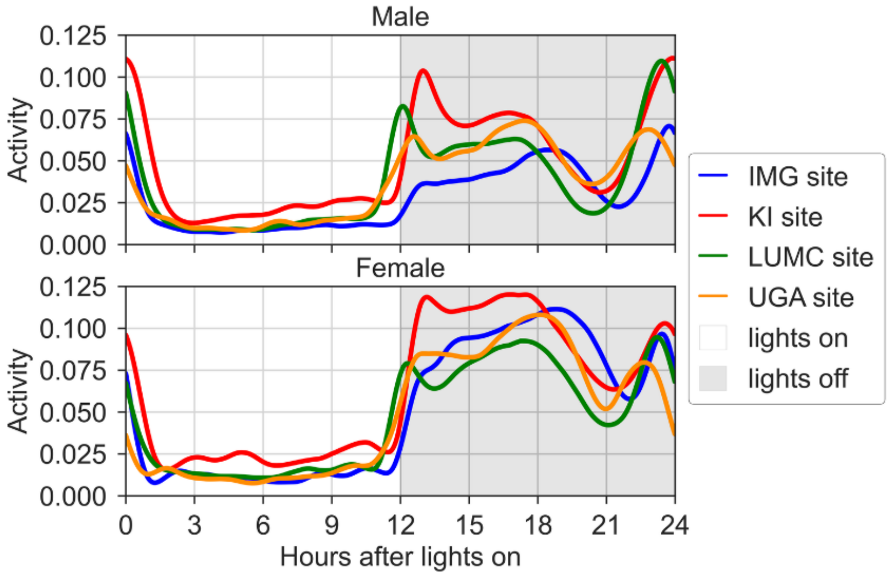

Fig. 2 Circadian rhythmicity of activity for male (a) and female (b) mice at the four sites. **a** and **b** show the average pattern of raw activity (average min^{-1}) for male (a) and female (b) mice during lights on (ZT 0-12, day) and light off (ZT 12-24, night) at the four participating sites. The circadian rhythm entrains to lights on and off and replicates closely across sites. Note the higher activity of female over male mice during lights-off.

Fig. 3

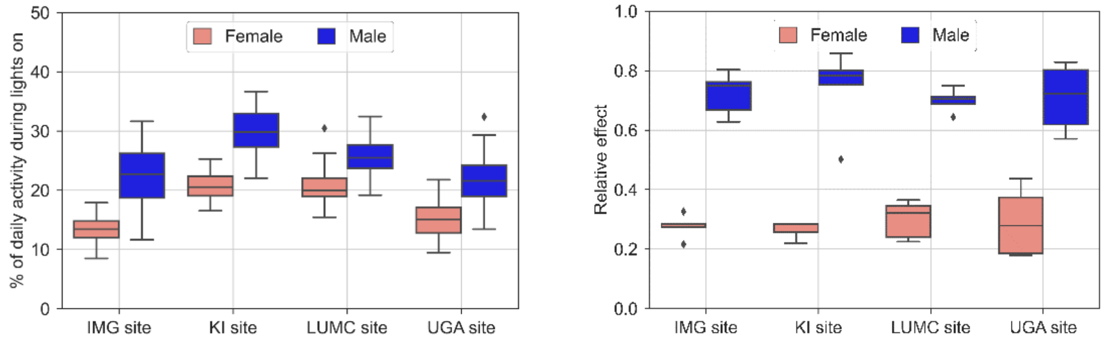

Fig. 3 a Box plots showing diurnality, which is the percentage of daily activity (%) occurring during lights on, within cage-change cycles, for male (blue) and female (pink) mice at the different sites. Note that males allocate more of the daily activity to the period with lights on, compared to females. **b** Relative effect size of of sex (male (blue) and female (pink)) on diurnality, across cage-change cycles, for mice at the different sites.

Fig. 4

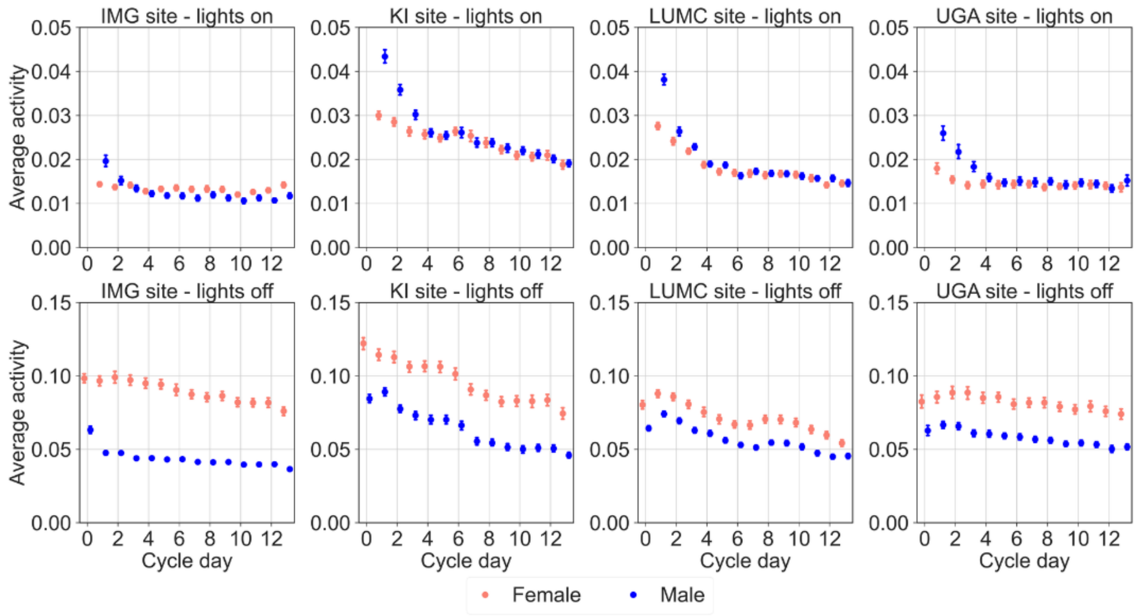

Fig 4 Activity during each day (upper row panels) and night (lower row panels) following a cage change. Average activity ± SEM, for all male (blue) and female (pink) cages, for each day (abscissa) of the period across the five cage change cycles at each site (columns of panels). For daytime, day 1 is the day that follows upon a cage change day while for night time day 0 is the night following the cage-change that took place during the preceding day time. Data was plotted separately by day and night for a better visualisation, but the light factor was tested as a repeated factor (subplot factor in nparLD testing).

Fig. 5

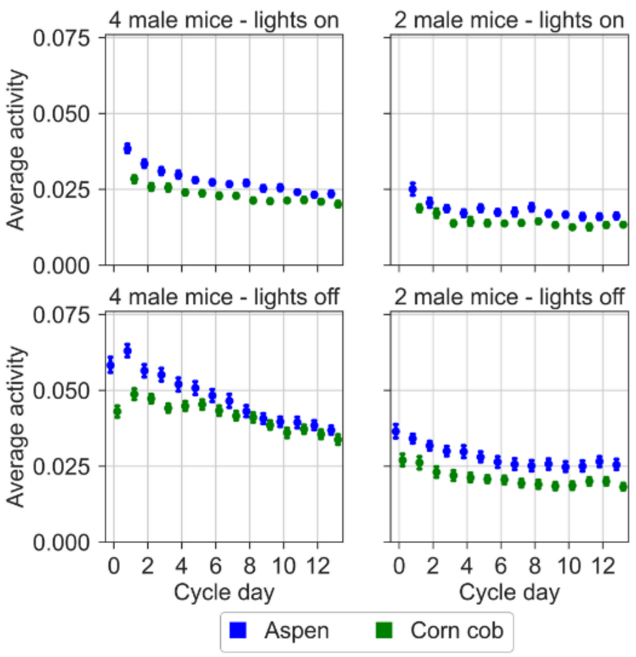

Fig. 5 Activity during each day (upper panels) and night (lower panels) periods, for n=4 (left two panels) and n=2 (right two panels) mice per cage, housed on aspen chips or corn cob (see the key to symbols). Data are average not-normalised activity values ± SEM. For daytime, day 1 is the day that follows upon a cage change day while for night time day 0 is the night that follows upon the cage-change which took place during the preceding day time (day 0).

Latrine prediction

As described in Materials and Methods, we used digital ventilated cages (DVC®, Tecniplast SpA) to determine the position of the latrine inside the cage, because DVC® capacitance readings are affected by the presence of water and therefore urine. In fact, we observe a drop of the signal of the electrodes below the latrine and its immediate surrounding, that increases over days as the latrine becomes larger. Therefore, for each cage and for each cage change cycle, we calculated the capacitive drop as the difference between the average of capacitance readings over each night of the cycle and the average of the first night of the cycle for each electrode. We then considered the data of the last night of the cycle to determine the latrine position as the area with the highest average drop (rear with electrodes 1-2-3-4-5-6, front with 7-8-9-10-11-12 electrodes). Fig. S5 and S6 show the capacitive drop in representative cages for each electrode and over days of a cage-change cycle.

Fig. 6

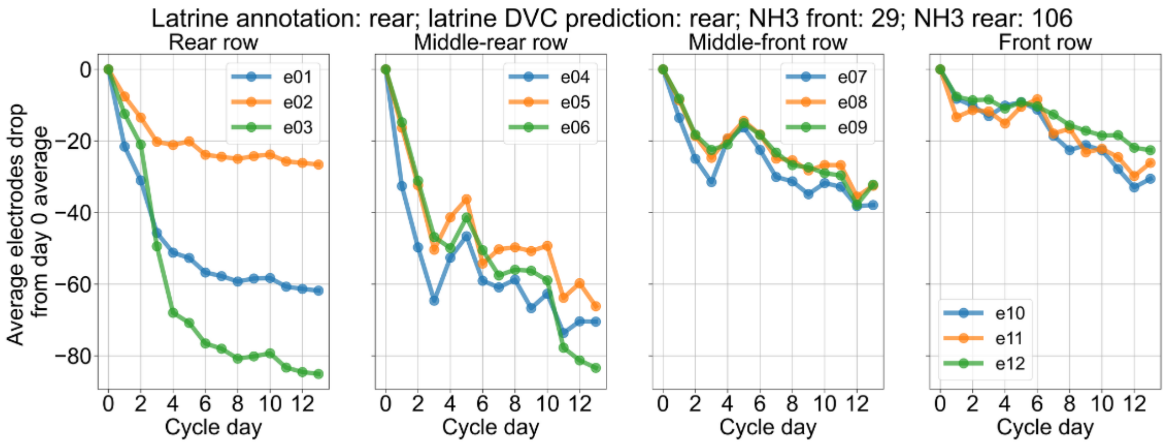

Fig. 6 Electrodes drop across days post cage change. The figure shows the progression of the capacitance drop of a representative cage of 4 male mice with the latrine developing in the rear as per visual inspection during a cage-change cycle. Each electrode drop was calculated as the difference with respect to the average capacitance value of the first night (day 0). The drop is higher in the latrine area in the rear section of the cage.

Figs 7-9

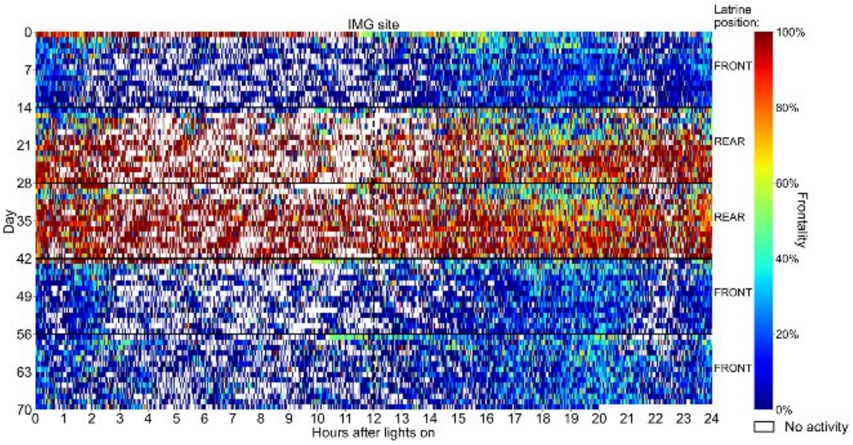

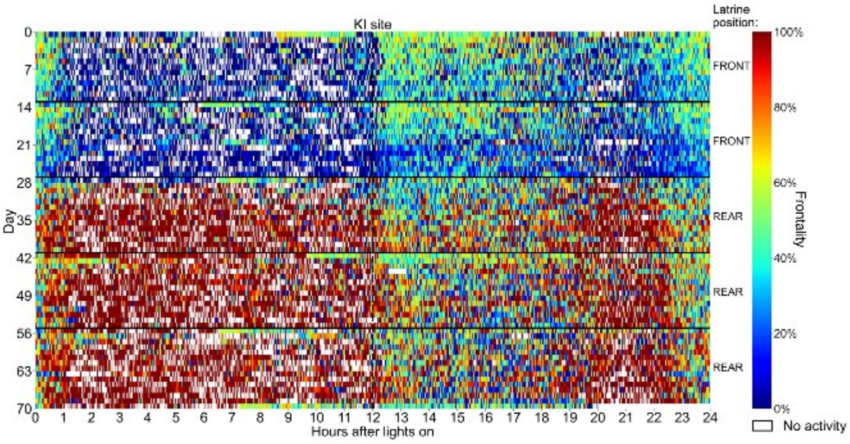

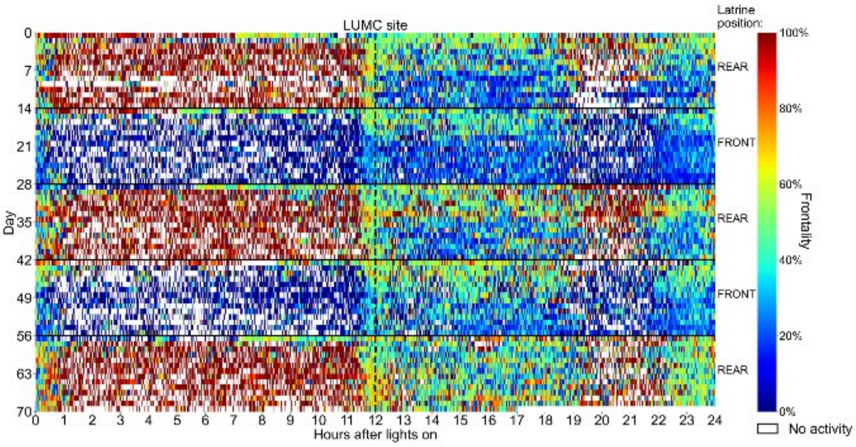

Figs. 7-9 Frontality and latrine positions across cage-change cycles at the (7) IMG, (8) KI and (9) LUMC sites. Heat maps showing for the five cage-change cycles, the frontality for each minute, of one example cage at each site. Frontality is the percentage of activity performed in the front-half of the cage-floor over the total activity performed on the entire cage floor, therefore red indicates that activity is taking place mostly in the front-half of the cage, while blue indicates that activity is taking place mostly in the rear. White denotes absence of activity (rest). Lights off at ZT 12 has been indicated by a dashed black vertical line. To the right is indicated the position of the latrine(s) of each

cycle. Note that placement of latrine(s) varies between cycles and that activity is more pronounced in the latrine free floor area, in particular during daytime when the animals rest. Furthermore, the pattern of allotting activity to the latrine-free area of the cage floor replicates well across sites.

Fig. 10

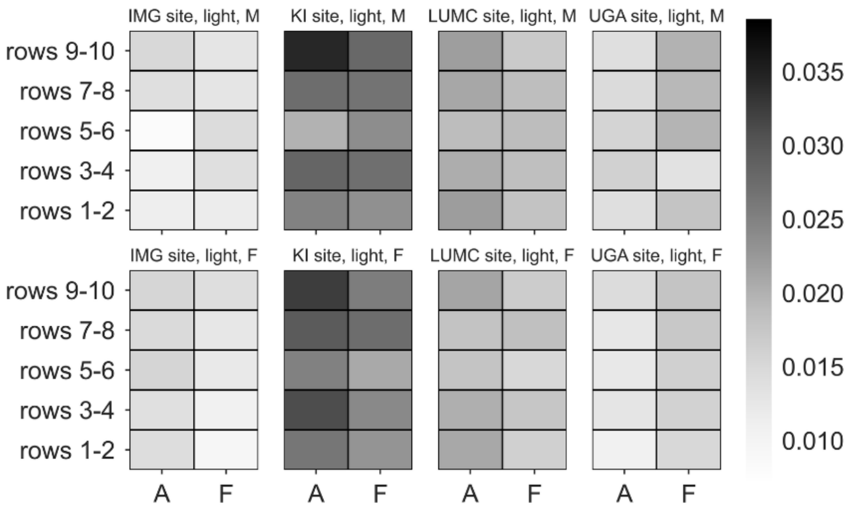

Fig. 10 Average activity for each row of cage positions housing female (F, even numbers; lower set of panels) and male (M, odd row number; upper set of panels) during lights on phase. Activity level is indicated by grey scale.

Fig. 11

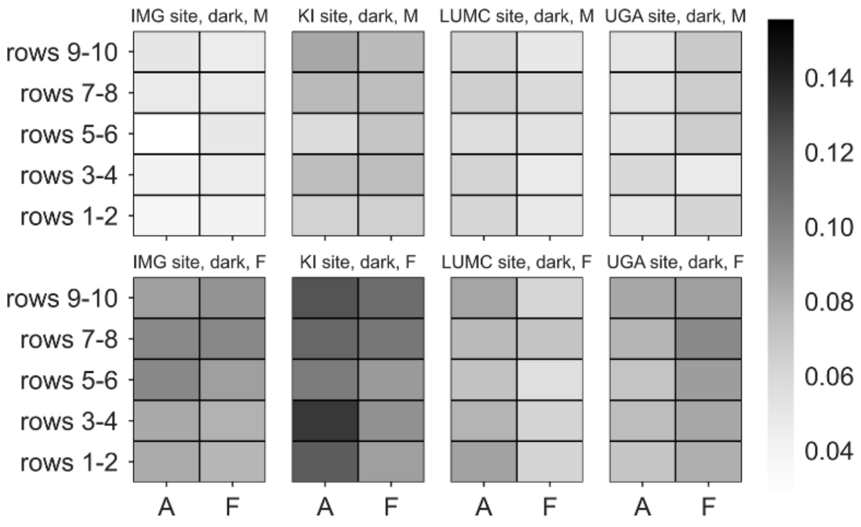

Fig. 11 Average activity for each row of cage positions housing female (F, even numbers; lower set of panels) and male (M, odd row number; upper set of panels) during lights-off phase. Activity level is indicated by grey scale.

Figs 12-14

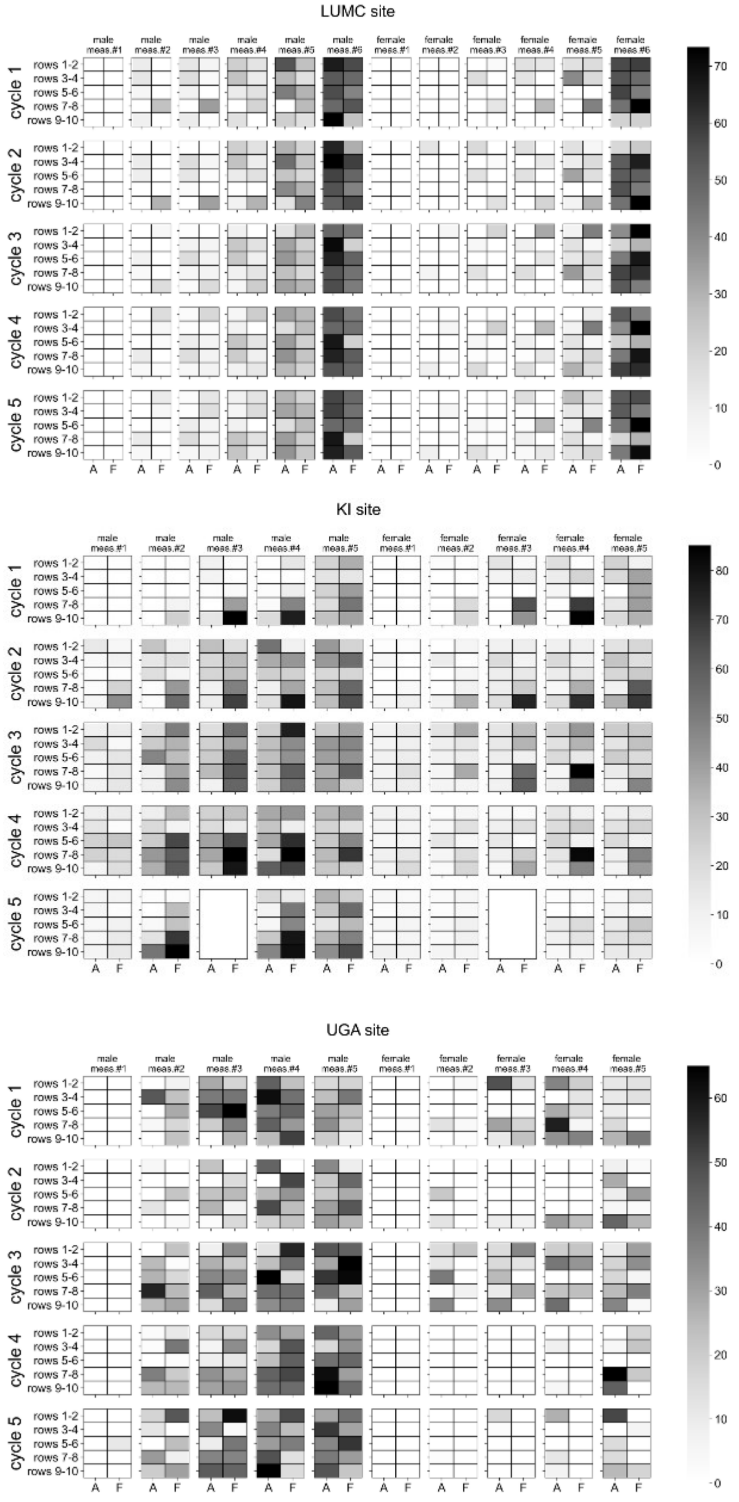

Figs. 12-14 Average NH_3 ppm in cages with male and female mice for each cage position and each cage change cycle at KI (12), LUMC (13) and UGA (14). For each subplot, on the abscissa is the lateral columns of the DVC rack (A is the first column on the left side, F the rightest column), on the ordinate is the row numbering from top to bottom of the rack at the two sites. Cages with male mice were rotated between rows with odd numbers while female cages were rotated between cages with even numbers (see Material and Methods). There is a cycle-to-cycle variability without any visible correlation between cage position and NH3 ppm.

Fig. 15

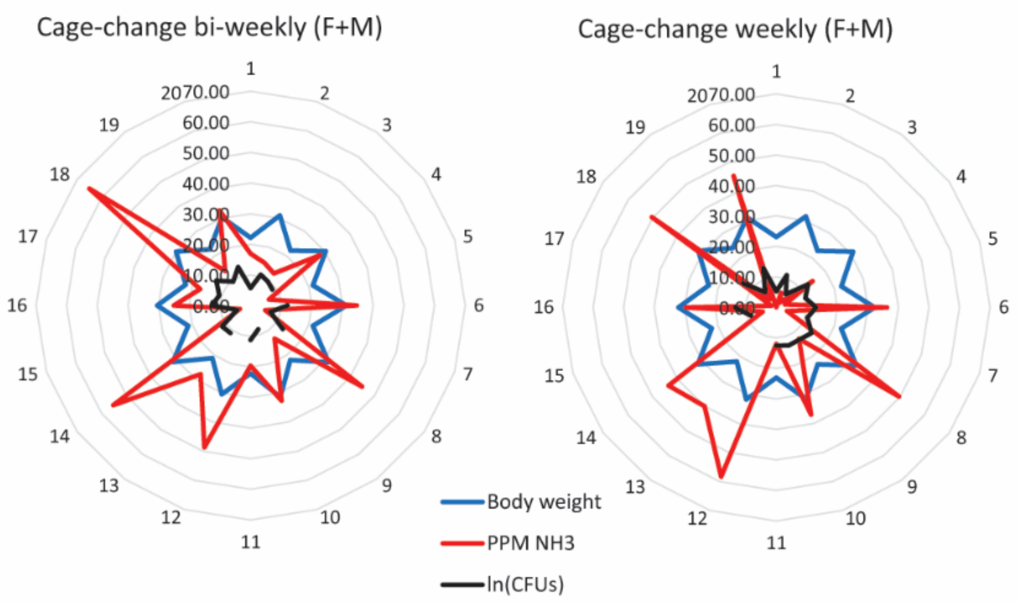

Fig. 15. A, B Diagrams showing the association between average body weight (BW), CFUs (lnCFUs) and NH_3 ppm for the 20 cages of female (uneven numbers of the circle) and male (even numbers of the circle) when cages were changed bi-weekly (A) and subsequently every week (B). Note that cages with higher NH3 ppm during the bi-weekly cage (#8, #12, #14 and #18 in A)-change also are the cages with higher values during weekly cage change (B).
